# Characteristics of Symptomatic Intracranial Hemorrhage in Patients Receiving Non-Vitamin K Antagonist Oral Anticoagulant Therapy

**DOI:** 10.1371/journal.pone.0132900

**Published:** 2015-07-14

**Authors:** Hisanao Akiyama, Kenji Uchino, Yasuhiro Hasegawa

**Affiliations:** Department of Neurology, St. Marianna University School of Medicine, Kawasaki, Kanagawa, Japan; IIBB-CSIC-IDIBAPS, SPAIN

## Abstract

**Objectives:**

The first non-vitamin K antagonist oral anticoagulant (NOAC) introduced to the market in Japan was dabigatran in March 2011, and three more NOACs, rivaroxaban, apixaban, and edoxaban, have since become available. Randomized controlled trials of NOACs have revealed that intracranial hemorrhage (ICH) occurs less frequently with NOACs compared with warfarin. However, the absolute incidence of ICH associated with NOACs has increased with greater use of these anticoagulants, and we wanted to explore the incidence, clinical characteristics, and treatment course of patients with NOACs-associated ICH.

**Methods:**

We retrospectively analyzed the characteristics of symptomatic ICH patients receiving NOACs between March 2011 and September 2014.

**Results:**

ICH occurred in 6 patients (5 men, 1 woman; mean ± SD age, 72.8 ± 3.2 years). Mean time to onset was 146.2 ± 111.5 days after starting NOACs. Five patients received rivaroxaban and 1 patient received apixaban. None received dabigatran or edoxaban. Notably, no hematoma expansion was observed within 24 h of onset in the absence of infusion of fresh frozen plasma, activated prothrombin complex concentrate, recombinant activated factor VIIa or hemodialysis. When NOAC therapy was initiated, mean HAS-BLED and PANWARDS scores were 1.5 ± 0.5 and 39.5 ± 7.7, respectively. Mean systolic blood pressure was 137.8 ± 15.9 mmHg within 1 month before spontaneous ICH onset.

**Conclusion:**

Six symptomatic ICHs occurred early in NOAC therapy but hematoma volume was small and did not expand in the absence of infusion of reversal agents or hemodialysis. The occurrence of ICH during NOAC therapy is possible even when there is acceptable mean systolic blood pressure control (137.8 ± 15.9 mmHg) and HAS-BLED score ≤ 2. Even stricter blood pressure lowering and control within the acceptable range may be advisable to prevent ICH during NOAC therapy.

## Introduction

Patients with cardiogenic embolism associated with nonvalvular atrial fibrillation often experience serious and recurrent embolic events. Anticoagulant therapy with warfarin has been used for a long time to prevent this condition, but warfarin also has various disadvantages, such as complexity of dosing, interaction with many foods and medications, and a high risk of hemorrhagic complications. To avoid these problems, dabigatran, indicated for “patients with nonvalvular atrial fibrillation for prevention of ischemic stroke and systemic embolism” [1], was first introduced to the Japanese market as a non-vitamin K antagonist oral anticoagulant (NOAC) in March 2011. Three more NOACs, rivaroxaban, apixaban, and edoxaban, have since become available. Although large-scale clinical studies showed advantages of NOACs over warfarin with regard to hemorrhagic complications [2–7], several cases of intracranial hemorrhage (ICH) associated with NOAC therapy have been reported with the increasingly widespread use of NOACs [8–22]. Nevertheless, the number of studies reporting such cases, especially cases of patients receiving rivaroxaban or apixaban, is still limited.

To date, we have treated 6 patients who developed symptomatic ICH while receiving NOAC therapy (rivaroxaban or apixaban). In this article, we discuss their clinical characteristics, focusing in particular on blood pressure control before ICH onset, and compare them with those of patients reported by other groups.

## Subjects and Methods

### Ethic Statement

The study was conducted in accordance with the guiding principles of the Declaration of Helsinki and was approved by the local ethics committee of St. Marianna University Hospital (No. 2877). All patients or their next of kin gave written informed consent to participate in this study.

### Patients and demographic data

Subjects were 6 consecutive patients admitted to our hospital between March 2011 (when dabigatran was launched in Japan) and September 2014 in whom symptomatic ICH occurred during NOAC therapy. Four NOACs are currently available in the Japanese market, but edoxaban, which was launched on 26 September 2014 at almost the end of this 3.5-year study, was not administered to any subject in this study.

The following clinical factors and outcome at the time of hospital discharge, as noted in the medical records and from reading brain computed tomography (CT) images, were examined retrospectively: age; sex; chief complaints and neurologic findings on hospital admission; underlying illness; type of NOAC; NOAC dose and duration of NOAC therapy; time between the last NOAC administration and ICH onset; concurrent anticoagulation; blood pressure on hospital admission and within 1 month before ICH onset; blood test results; predictive scores for the risk of cerebral infarction (CHADS_2_) and bleeding (HAS-BLED and PANWARDS); and ICH-related information (location, hematoma volume, hematoma expansion, and surgical treatment). Our findings were then compared with corresponding results in the recent literature.

### Statistical analysis

Welch’s t-test was used to compare the means calculated in this retrospective study and those obtained from the results reported previously by other groups. Creatinine clearance rate was calculated using the Cockcroft-Gault equation. Hematoma volume was calculated with the following formula using measurements on a plain CT image of the brain: maximum length of an ellipsoid representing intraparenchymal hemorrhage × width perpendicular to maximum length × thickness of hematoma × 0.5. Hematoma expansion was defined as volume increasing by > 6 ml or > 33% [23–24]. Mean annual incidence rates of ICH were calculated by the person-years method. Results with p value less than 0.05 were considered statistically significant.

## Results

### Clinical background (Table 1)

**Table 1:** Clinical characteristics of our 6 cases (1). NOAC, Non-vitamin K antagonist oral anticoagulant; mRS, Modified Rankin Scale; M, Male; F, Female; IC, Impaired consciousness; Ri, Rivaroxaban; Ap, Apixaban; ASA, Acetylsalicylic acid; Rt, Right; Lt, Left; Th, Thalamus; SAH, Subarachnoid hemorrhage; SDH, Subdural hemorrhage; Ca, Caudate head hemorrhage; Pu, Putamen; ICH, Intra cerebral hemorrhage.

Present study	Case 1	Case 2	Case 3	Case 4	Case 5	Case 6
Age (years)	69	73	73	78	75	69
Gender	M	M	F	M	M	M
Chief complain	IC	IC	IC	Motor paresis in the right lower extremity	Sensory impairment in the right upper extremity	Dysarthria
Type of NOACs	Ri	Ri	Ap	Ri	Ri	Ri
Daily dose of NOAC (mg)	15	10	10	10	10	10
Duration of NOAC therapy (days)	124	54	99	51	171	378
Time between the last NOAC administration and ICH onset (hours)	12	Unknown	10	12	4	10
Antiplatelet therapy	-	+ (ASA 100mg)	-	-	+ (ASA 100mg)	-
Hypertension	+	-	+	+	+	+
Diabetes mellitus	-	-	-	-	+	-
Dyslipidemia	-	-	+	+	+	+
Location of hematoma	Rt. Th	SAH with bil. SDH	Rt. Ca	Lt. Th	Lt. Th	Rt. Pu
Ventricular rupture	+	-	+	-	-	-
Hydrocephalus	+	-	+	-	-	-
Hematoma volume (ml)	13	-	-	1.38	1.8	3.56
Hematoma expansion	-	-	-	-	-	+
Pre-morbid mRS	0	0	0	0	0	4
Head injury	-	+	-	-	-	-
Operation	+	-	+	-	-	-
CHADS_2_ score	1	1	3	4	3	1

We encountered 6 patients (5 men [83.3%], 1 woman; mean age at onset, 72.8 ± 3.2 years; age range, 69–78 years) who developed symptomatic ICH while receiving NOAC therapy during the 3.5-year study period between March 2011 and September 2014. Three patients showed impaired consciousness. Five patients (83.3%) had the same underlying condition (hypertension) and were receiving antihypertensive therapy. In addition, 1 patient (16.7%) had diabetes mellitus and 4 (66.7%) had dyslipidemia.

The most common NOAC given to patients in this study was rivaroxaban, which was given to 5 patients (83.3%); the remaining patient received apixaban. None of the patients received dabigatran. Mean daily dose of rivaroxaban was 11.0 ± 2.0 mg (10 mg in 4 patients, 15 mg in 1 patient). In Japan, the recommended dose of rivaroxaban is 15 mg orally once daily, and if creatinine clearance rate is 15 to 50 ml/min, a dose reduction to 10 mg orally once daily is recommended. The daily dose of apixaban was 10 mg in the remaining patient. Mean interval between initiation of NOAC therapy and onset of symptomatic ICH was 146.2 ± 111.5 days (range, 51–378 days), and mean time between the last NOAC administration (known for 5 patients only) and ICH onset was 9.6 ± 2.9 h (range, 4–12 h). Two patients received concurrent antiplatelet therapy with 100 mg/day aspirin.

### ICH-related information (Tables 1 and 2, Fig 1)

**Table 2:** Clinical characteristics of our 6 cases (2). BMI, Body mass index; BP, Blood pressure; ICH, Intra cerebral hemorrhage; APTT, Activated partial thromboplastin time; PT-INR, prothrombin time-international normalized ratio; Ccr, Creatinine clearance; NOAC, Non-vitamin K antagonist oral anticoagulant; mRS, Modified Rankin Scale; NIHSS, National Institute of Health Stroke Scale; NSAID, Non-steroidal anti-inflammatory drug; Ap, Apixaban; Wa, Warfarin; Tr, Transferred to the rehabilitation hospital; D, Death; Cd, Changed departments; NE, Not evaluated.

Present study	Case 1	Case 2	Case 3	Case 4	Case 5	Case 6
Body weight (kg)	50.7	64.1	66.5	64.7	70.5	49
BMI	17.1	23	28.3	27.1	25.9	20.9
Platelet on admission (×10^4^/μl)	28	12.7	22.4	14.3	26.6	17.4
BP on admission (mmHg)	184/76	147/101	169/122	148/82	162/56	131/78
BP within 1 month of ICH onset (mmHg)	147/92	63/45	118/67	152/81	153/66	119/67
APTT (sec)	28.9	36.9	37.2	42.2	35.5	42.5
PT-INR	0.98	1.51	1.11	1.61	1.19	1.52
Ccr on admission (ml/min)	82	31	73	58	70	48
Ccr on NOACs introduction (ml/min)	80	41	51	55	63	46
NIHSS score on admission	16	NE	NE	1	10	5
Liver abnormality	-	-	-	-	-	-
NSAIDs	-	-	-	-	-	-
HAS-BLED score	1	2	1	1	2	2
PANWARDS score	41	30	39	55	37	35
Post mRS	4	6	3	1	1	4
Hospital stay (days)	90	-	35	34	18	55
Final anti-thrombotic therapy	-	-	Ap	Wa	Ap	Ap
Outcome	Tr	D	Cd	Tr	Tr	Tr

**Fig 1:**
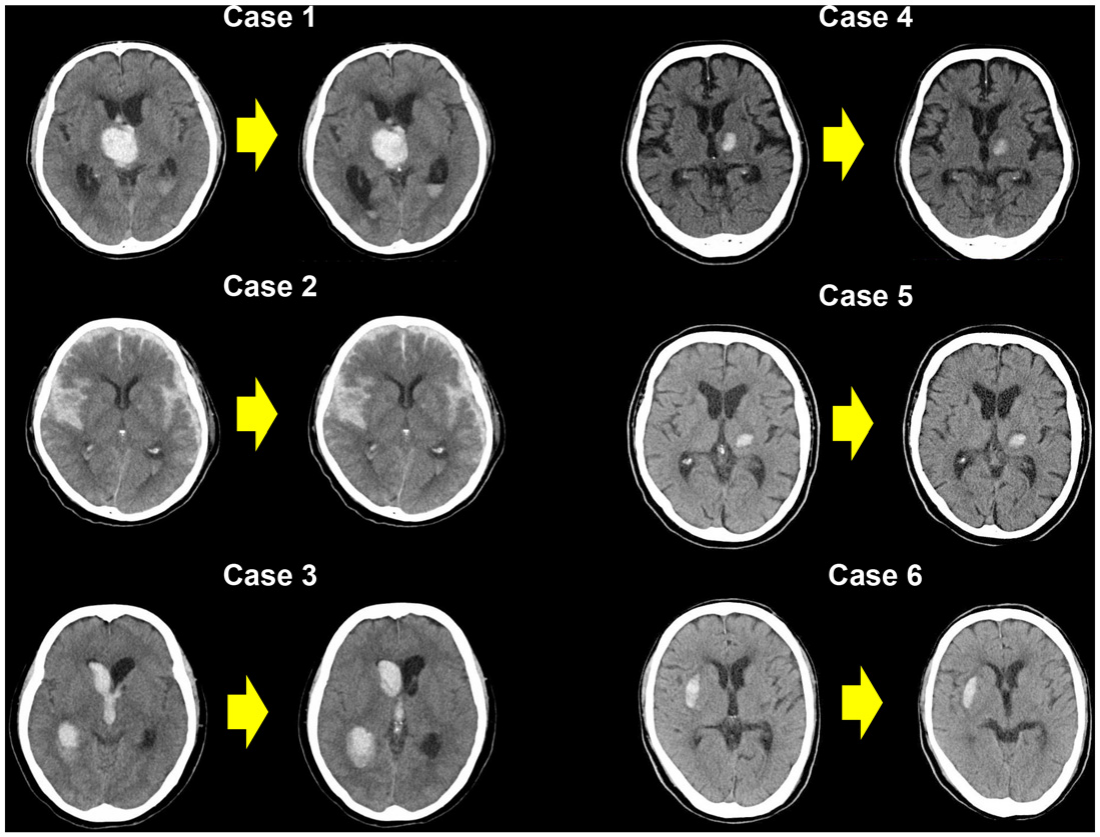
Brain computed tomography (CT) on admission (left) and 24 h later (right) in Cases 1, 3–6, 1.5 h later (right) in Case 2 who had intracranial hemorrhage during NOAC therapy. No hematoma expansion is evident in any of the cases.

ICHs were located in the parenchyma in most cases (5/6, 83.3%): 3 in the thalamus, 1 in the putamen, 1 in the caudate head, and 1 in the subarachnoid with bilateral subdural hematoma. The median modified Rankin scale (mRS) score was 0 (range, 0–4). Traumatic ICH was suspected in 1 patient, who developed subarachnoid hemorrhage with bilateral subdural hematoma while receiving rivaroxaban therapy (Case 2). Two patients (33.3%) underwent surgery (endoscopic hematoma evacuation) for obstructive hydrocephalus as a complication of hematoma but not for hematoma expansion.

Hematoma volume measured on head CT images was small in 4 patients (Cases 1, 4–6), with a mean estimated volume of 4.9 ± 4.7 ml. Cases 2 and 3 were excluded as the hematoma could not be measured because of its form. Hematoma expansion within a 24-h period was not observed in Cases 1, 3–6; Case 2 died before receiving inpatient treatment. None of the 6 patients received an infusion of fresh frozen plasma (FFP), activated prothrombin complex concentrate (PCC), recombinant activated factor VIIa (rFVIIa) or hemodialysis. Hematoma volume on hospital admission was very small (1.38 ml) in Case 4, even though oral rivaroxaban therapy was continued for 4 days after the onset of motor paresis possibly due to ICH.

In the 6 patients, mean age at initiation of NOAC therapy was 72.7 ± 3.4 years, mean body weight was 60.9 ± 8.1 kg (range, 49.0–70.5 kg), and mean BMI was 22.3 ± 3.9. There were no problems with initiation of NOAC therapy in any cases. In 2 of the 5 patients receiving rivaroxaban, the daily dose was appropriately reduced to 10 mg because of a creatinine clearance rate of 30–49 ml/min, while in the remaining 3 patients, the dose was inappropriately reduced for 2 patients whose creatinine clearance rate (≥ 50 ml/min) did not meet the dose reduction criteria. The dose of apixaban was 10 mg daily in the 1 patient that took it in this study, which was appropriate since his age, body weight, and serum creatinine clearance rate did not meet the dose reduction criteria.

Tests of blood specimens collected upon hospitalization (at ICH onset) showed a mean platelet count of 202,000 ± 59,000 cells, mean activated partial thromboplastin time (APTT) of 37.2 ± 4.6 s, prothrombin time-international normalized ratio (PT-INR) of 1.32 ± 0.24 and creatinine clearance rate of 60.3 ± 17.0 ml/min. The creatinine clearance rate was also favorable (56.0 ± 12.8 ml/min) at initiation of NOAC therapy. None of the patients abused alcohol or had hepatic damage. None received non-steroidal anti-inflammatory drugs (NSAIDs).

Mean systolic and diastolic blood pressure was high at admission (156.8 ± 17.1 mmHg and 85.8 ± 20.8 mmHg, respectively). Within 1 month before ICH onset, the former was 125.3 ± 31.4 mmHg (range, 63–153 mmHg) and the latter was 69.7 ± 14.5 mmHg (range, 45–92 mmHg), suggesting reasonable blood pressure control in all patients. However, in the 5 patients with spontaneous ICH (excluding Case 2, in which traumatic ICH occurred), mean systolic and diastolic blood pressure was moderately high (137.8 ± 15.9 and 74.6 ± 10.3 mmHg, respectively; range 118–153 / 66–92 mmHg) albeit within the acceptable range.

HAS-BLED and PANWARDS scores, which predict the risk for intracranial bleeding, were 1.5 ± 0.5 (median 1.5) and 39.5 ± 7.7, respectively, at initiation of NOAC therapy, suggesting a low risk in all 6 patients.

Five of the 6 patients received NOAC therapy at our hospital and the remaining patient received it initially at our hospital and then at the nearby clinic. The total number of prescriptions of each NOAC at our hospital was 171 dabigatran prescriptions in 43 months, 342 rivaroxaban prescriptions in 30 months, and 233 apixaban prescriptions in 20 months. Under the assumption that all prescriptions were provided by our hospital, the annual incidence rate of symptomatic ICH was found to be higher with rivaroxaban (0.58%) than with dabigatran (0%) or apixaban (0.26%). The overall annual incidence rate of NOACs-associated ICH was 0.22% (746 prescriptions in 43 months).

### Outcome at discharge (Table 2)

The mRS score at the time of discharge was 1 in 2 patients, 3 in 1 patient, and 4 in 2 patients. The median score in 5 patients was 3 (excluding deceased Case 2). Case 6 had the mRS score of 4 at discharge and also before ICH onset, suggesting that there were few cases of disease aggravation.

One patient was transferred to the hematology department after being diagnosed with diffuse large B-cell lymphoma. When the number of days of stay in our department was used for calculation, mean duration of hospital stay was 46.4 ± 24.8 days (range, 18–90 days). One patient required a long hospital stay (90 days) because of ventriculoperitoneal shunting for aqueduct stenosis and treatment for a complication (Methicillin-Resistant Staphylococcus Aureus pneumonia). Four patients were eventually transferred to rehabilitation clinics.

After discharge, 1 patient did not receive anticoagulant therapy and 4 received anticoagulant therapy with a NOAC that was different from the one given before ICH onset. Previously used NOACs were changed to warfarin in 1 patient and apixaban in 3 patients.

## Discussion

Warfarin has been used worldwide for more than 50 years in anticoagulant therapy for the prevention of cardiogenic embolism associated with nonvalvular atrial fibrillation. However, NOACs are gradually replacing warfarin after several randomized controlled trials (RCTs) conducted in recent years (RE-LY, ROCKET AF, J-ROCKET AF, ARISTOTLE, AVERROES, and ENGAGE AF) showed that NOACs are clinically equivalent or superior to warfarin in reducing the risks of cerebral infarction and ICH [2–7]. In Japan, dabigatran was launched in March 2011, rivaroxaban in April 2012, and apixaban in February in 2013, and edoxaban was approved for additional indications in September 2014. NOACs are known to have a lower risk of ICH complications than warfarin, but recent studies, albeit with a limited number of cases, have demonstrated the occurrence of ICH associated with NOAC therapy as the use of NOACs has become widespread [8–22].

The clinical characteristics in these previous studies (Tables 3 and 4) were as follows [8–22]. Mean age at ICH onset while receiving NOAC therapy was 79.3 ± 6.6 years, which was significantly higher (p = 0.003) than in our study. The number of male patients was higher than that of female patients. Dabigatran tended to be given in cases of subdural hemorrhage, while rivaroxaban tended to be given in cases of intraparenchymal hemorrhage. None of the previously reported patients received apixaban. Mean duration of NOAC therapy was 102.2 ± 114.6 days. Some patients were also receiving concurrent antiplatelet therapy, and hypertension was observed in most cases. Hematoma volume was small and remained so. Using the blood test results and clinical data (e.g., blood pressure) available, we calculated that mean systolic and diastolic blood pressure at ICH onset was 159.8 ± 28.7 mmHg and 90.4 ± 16.5 mmHg, respectively. Blood pressure before ICH onset was not available in any of the previously reported cases.

**Table 3:** Characteristics of symptomatic ICH in patients receiving NOACs (1). mRS, Modified Rankin Scale; NOAC, Non-vitamin K antagonist oral anticoagulant; BP, Blood pressure; ICH, Intracerebral hemorrhage; Cr, Creatinine; eGFR, Estimate glomerular filtration rate; Ccr, Creatinine clearance; PT-INR, Prothrombin time-international normalized ratio; APTT, Activated partial thromboplastin time; M, Male; F, Female; Sub, Subcortical hemorrhage; SDH, Subdural hemorrhage; SAH, Subarachhnoid hemorrhage; Th, Thalamic hemorrhage; Bg, Basal ganglia hemorrhage; NR, Not reported; Da, Dabigatran; Ri, Rivaroxaban; S, small; L, Large; m, Month; w, Week; d, Day; MF, Mechanical fall; HT, Head trauma; BE, Burr-hole evacuation; D, Death; Dh, discharged to home; Tr, Transferred to the rehabilitation hospital.

Past reports	2012 Garber et al	2012 Chen et al	2013 Chang et al	2013 Awad et al	2013 Wassef et al	2014 Faust et al	2014 Simonsen et al	2014 Kasliwal et al	2014 Ross et al	2014 Hana et al
Number of cases	1	1	1	1	3	1	1	2	7	4
Age (years)	83	80	94	85	79/72/72	85	75	83/80	NR/NR/NR/NR/NR/NR/NR	77/73/78/NR
Gender	M	M	M	F	F/M/M	M	M	M/M	NR/NR/NR/NR/NR/NR/NR	M/F/M/M
Location of hematoma	Sub+SDH+SAH	SDH	SDH	SDH	Traumatic Sub/SDH/SAH	Bg+Th	Sub	Th/SDH	ICH/ICH/ICH/ICH/ICH/ICH/ICH	Th/Sub/Th/Sub
Hematoma volume (ml)	NR	S	L	2.2cm	NR/NR/NR	2.5cm in diameter	15	2×1cm/NR	NR/NR/NR/NR/NR/NR/NR	S/L/NR/S
Hematoma expansion	+	-	-	+	+ / - /-	+	+	- /-	NR/NR/NR/NR/NR/NR/NR	+ /NR/NR/ +
Pre—morbid mRS	NR	NR	NR	NR	NR/NR/NR	NR	NR	NR/NR	NR/NR/NR/NR/NR/NR/NR	NR/NR/NR/NR
Type of NOACs	Da	Da	Da	Da	Da/Da/Da	Da	Da	Ri/Da	Da/Da/Da/Da/Da/Da/Da	Ri/Ri/Da/Da
Duration of NOAC therapy (days)	1m	NR	7m	NR	NR/NR/NR	2w	54d	NR/NR	NR/NR/NR/NR/NR/NR/NR	NR/2w/NR/NR
Daily dose of total NOACs (mg)	300	300	300	NR	300/300/NR	300	300	20/300	Most 300	NR/NR/NR/NR
Anti-thrombotic therapy	NR	NR	NR	NR	- / + / -	NR	NR	NR/NR	NR/NR/NR/NR/NR/NR/NR	NR/NR/NR/NR
Hypertension	NR	NR	NR	NR	- / + / -	+	+	+ /NR	NR/NR/NR/NR/NR/NR/NR	+ / + / + /NR
Head injury	+ (MF)	+ (MF)	+ (MF)	+ (HT)	+ (MF)/ - /+ (MF)	-	NR	NR/NR	NR/NR/NR/NR/NR/NR/NR	NR/NR/NR/NR
BP on admission (mmHg)	NR	149/64	NR	NR	NR/NR/NR	182/98	138/76	NR/NR	NR/NR/NR/NR/NR/NR/NR	NR/NR/NR/NR
BP within 1 month of ICH onset (mmHg)	NR	NR	NR	NR	NR/NR/NR	NR	NR	NR/NR	NR/NR/NR/NR/NR/NR/NR	NR/NR/NR/NR
Cr on admission (mg/dl)	NR	1.31	0.74	NR	NR/NR/NR	0.9	NR	Normal/Normal	NR/NR/NR/NR/NR/NR/NR	NR/NR/NR/NR
eGFR on admission (ml/min/1.73m^2^)	NR	NR	79	NR	>90/>90/46	NR	NR	NR/NR	NR/NR/NR/NR/NR/NR/NR	NR/NR/NR/NR
CCr on admission (ml/min)	NR	53.69	NR	NR	NR/NR/NR	NR	71	NR/NR	NR/NR/NR/NR/NR/NR/NR	NR/NR/NR/NR
PT-INR on admission	1.4	1.2	1.52	NR	Normal/1.2/2.0	NR	1.3	Normal/1.48	NR/NR/NR/NR/NR/NR/NR	2.5/2.3/Normal/NR
APTT on admission (sec)	43	37.6	39.8	NR	Normal/26/47	45.7	59	Normal/45.8	NR/NR/NR/NR/NR/NR/NR	NR/NR/NR/NR
Platelet on admission (×10^4^/μl)	27.1	24.7	NR	NR	NR/18.3/11.9	NR	NR	NR/NR	NR/NR/NR/NR/NR/NR/NR	NR/NR/NR/NR
Operation	-	-	+ (BE)	+ (BE)	- / + (BE)/-	-	-	- / +	Total 3 cases	+ / - / - /-
Outcome	D	Tr	Tr	Dh	D/Dh/Dh	Tr	D	Dh/Tr	D/D/D/Dh/Dh/Dh/Tr	Tr/D/Tr/D

**Table 4:** Characteristics of symptomatic ICH in patients receiving NOACs (2). mRS, Modified Rankin Scale; NOAC, Non-vitamin K antagonist oral anticoagulant; BP, Blood pressure; ICH, Intracerebral hemorrhage; Cr, Creatinine; eGFR, Estimate glomerular filtration rate; Ccr, Creatinine clearance; PT-INR, Prothrombin time-international normalized ratio; APTT, Activated partial thromboplastin time; M, Male; F, Female; S, Small; Me, Medium; d, day; m, month; y, year; SDH, Subdural hemorrhage; SAH, Subarachhnoid hemorrhage; Th, Thalamic hemorrhage; Pu, Putaminal hemorrhage; Ce, Cerebellar hemorrhage; Bg, Basal ganglia hemorrhage; Ca, Caudate head hemorrhage; Sub, Subcortical hemorrhage; NR, Not reported; Da, Dabigatran; Ri, Rivaroxaban; MF, Mechanical fall; HT, Head trauma; BE, Burr-hole evacuation; VE, Ventricular drainage; D, Death; Dh, discharged to home; Tr, Transferred to the rehabilitation hospital.

Past reports	2013 Komori et al	2014 Hagii et al	2014 Shoji et al	2015 Debata et al	Total
Number of cases	8	5	5	3	43
Age (years)	79/87/80/86/74/87/92/82	67/80/75/82/64	83/76/69/82/87	71/76/80	Mean 79.3 ± 6.6 (p = 0.003, compared to our 6 cases)
Gender	M/F/F/M/M/F/F/M	M/M/F/M/M	M/M/M/F/M	M/M/M	M; 27 F; 9
Location of hematoma	SDH/SDH/SDH/SDH/SDH/Traumatic SAH/Th/Pu	Th/Th/Ce/Th/Bg	SDH/Pu/Pu/Traumatic ICH/SDH	Th/Ca/Th	SDH; 13 SAH; 3 Sub; 5 Pu; 3 Th; 10 Bg; 2 Ce; 1
Hematoma volume (ml)	S/Me/Me/Me/Me/S/Me(5ml)/S(1ml)	10/4/5/1/2	NR/NR/Max diameter 4cm/NR/NR	2010/3/15	
Hematoma expansion	- / - / - / - / - / - / - /-	- / - / - / - / -	NR	- / + / +	+; 9
Pre- morbid mRS	0/0/0/0/0/0/3/0	0/4/1/3/0	0/1/0/0/0	NR	
Type of NOACs	Da/Da/Da/Da/Da/Da/Da/Da	Ri/Ri/Ri/Ri/Ri	Da/Da/Da/Da/Da	Ri/Ri/Ri	Da; 32 Ri; 11
Duration of NOAC therapy	1m/6m/3m/1m/8d/10m/9m/3m	29d/77d/>1y/>1y/14d	1m/2m/1m/1m/1m	NR	Mean 102.2 ± 114.6
Daily dose of total NOACs (mg)	220/220/220/220/220/220/220/220	15/10/15/10/15	220/220/300/150/220	15/15/10	Da; Mean 251.0 ± 46.0 Ri; Mean 13.9 ± 3.3
Antipletelet therapy	- / - / - / - / - / - / - / +	- / - / - / - / -	- / - / + / - /-	- / - /-	+; 3
Hypertension	+ / - / - / + / - / - / + / +	+ / + / + / + / +	+ / + / + / - /-	+ / + / +	+; 22
Head injury	- / + / + / + / + / + / - /-	NR	- / - / - / + /-	- / - /-	+; 12
BP on admission (mmHg)	100/75 120/60 156/89 123/69 130/79 149/84 164/85 154/90	176/115 162/95 167/110 168/93 163/102	158/82 215/126 201/92 148/88 221/123	141/98 209/96 151/81	Mean 159.8 ± 28.7/90.4 ± 16.5
BP within 1 month of ICH onset (mmHg)	126/70 104/60 112/81 129/72 124/82 128/61 NR 140/86	NR	NR	NR	Mean 123.3 ± 11.0/73.1 ± 9.5
Cr on admission (mg/dl)	NR	NR	1.08/1.05/0.98/0.68/1.23	- / - /1.19	Mean 1.02 ± 0.20
eGFR on admission (ml/min/1.73m^2^)	NR	NR	NR	NR	
CCr on admission (ml/min)	72.9/46/32/43/53/35/38/55	110/53/114/51/101	51/57/73/50/40	74/100.4/41.5	Mean 61.5 ± 23.7
PT-INR on admission	NR	0.90/1.27/0.92/0.97/1.15	1.31/NR/1.09/NR/1.11	1.02/1.17/1.14	Mean 1.35 ± 0.43
APTT on admission (sec)	31.6/47.2/46.9/37.5/38.2/45.8/44.7/72.4	34.0/36.4/29.5/36.5/38.1	39.2/45.8/36.9/38.0/34.4	28.9/32.4/29.1	Mean 40.3 ± 9.3
Platelet on admission (×10^4^/μl)	NR	NR	15.6/18.4/13.3/13.0/21.5	Normal/Normal/8.4	Mean 17.2 ± 5.6
Operation	- / + (BE) / + (BE) / + (BE) / + (BE) / - /-	- / - / - / - / -	+ (BE) / - / - / - /-	- / + / +	+; 15
Outcome	mRS 0/0/0/0/0/0/4/1	mRS 2/4/3/4/1	mRS 1(Dh)/3/4(Tr)/5/6	Tr/D/D	

In our study, we examined the clinical characteristics, including blood pressure control before ICH onset, of 6 consecutive patients admitted to our hospital with symptomatic ICH that occurred during NOAC therapy. We found that hematoma volume was small, onset occurred relatively early after initiation of NOAC therapy (≤ 6 months), and none of the patients showed expansion of a small hematoma in the absence of infusion of FFP, PCC, rFVIIa or hemodialysis. Our study suggests that infusion of these reversal agents or hemodialysis is not necessary to prevent hematoma expansion, but there is still debate about whether these agents are useful for preventing hematoma expansion or improving outcome [8–18, 22].

The advantage of NOACs is probably that they do not inhibit the production of factor VII and prothrombin [18–19, 21–22, 25], the key factors in the blood coagulation cascade, and they inhibit the coagulation activities of factor Xa and thrombin in a reversible manner, unlike warfarin. Another suggested reason is the loss of anticoagulant action of NOACs at blood trough concentrations, so that the intracranial hemostatic mechanism becomes normal temporarily. Several reasons for the differences in incidence of ICH among NOACs have been suggested but not proven; for example, differences in their dose, differences in peak and trough concentrations attributed to the dosage (e.g., once a day or twice a day), involvement of the thrombin-thrombomodulin complex, differences in tissue distribution volumes, involvement of apixaban-specific intestinal excretion, and differences in inhibition of MMP-9 activity shown in an animal model [26]. In the present study, mean time between onset of ICH and last NOAC administration was 9.6 h, suggesting the occurrence of hemorrhage irrespective of the peak blood levels of NOACs. This observation supports Kaneko et al.’s finding that peak blood levels were comparable between the groups with and without hemorrhage [27], suggesting the importance of blood pressure management during NOAC therapy (see below).

HAS-BLED proposed by Pisters et al. [28], PANWARDS by Hankey et al. [29], and Q-bleeds by Hippisley-Cox and Coupland [30] are commonly used scoring systems for prediction of hemorrhage during anticoagulant therapy. However, hemorrhage occurred in all patients in this study, despite the risk of hemorrhage being predicted as low by the above scores (HAS-BLED score ≤ 2, and PANWARDS score = 39.5 points, predicting the hemorrhage frequency of 2.5% within 2.5 years). It is well known that hypertension is the strongest risk factor for ICH, and indeed the HAS-BLED bleeding score was higher by 1 point in patients with poorly controlled blood pressure than in those with successful control (≤ 160 mmHg). We focused on blood pressure control before ICH onset during NOAC therapy in the present study because there was no strict target blood pressure level in previous studies. Blood pressure on admission was high in all the patients in our study, as expected at ICH onset (i.e., in the acute phase). However, we found that systolic blood pressure of 137.8 ± 15.9 mmHg within 1 month before spontaneous ICH onset when the case of traumatic subdural hematoma was excluded, suggesting a need for stricter blood pressure lowering and control. A few previous studies have investigated the target systolic blood pressure level during antithrombotic therapy, such as antiplatelet or warfarin therapy. In the BAT study [31], which was a prospective, multicenter, observational study, Toyoda et al. estimated that the optimal blood pressure level was < 130/81 mmHg among 4009 patients receiving oral antithrombotic therapy, not including NOACs (e.g., receiving single or dual antiplatelet therapy, warfarin therapy, or warfarin plus antiplatelet therapy). In the Perindopril Protection Against Recurrent Stroke (PROGRESS) study [32], Arima et al. found that blood pressure may need to be controlled at < 120 mmHg among 4876 patients receiving antithrombotic therapy for a mean duration of 3.9 years. In the SPS3 randomized trial [33], Benavente et al. found that the rate of ICH was significantly reduced (hazard ratio, 0.37; 95% confidence interval (95% CI), 0.15–0.95; p = 0.03) in the lower target blood pressure group (< 130 mmHg) among 3020 patients with recent lacunar stroke who took aspirin or aspirin with clopidogrel for a mean duration of 3.7 ± 2.0 years. In a subanalysis of the Cilostazol Stroke Prevention Study 2 (CSPS 2) [34], Uchiyama et al. found that cilostazol was associated with a significantly lower risk for hemorrhagic stroke compared with aspirin at all systolic blood pressure control levels (< 130, 130–140, ≥ 140 mmHg) and that the effect was particularly notable in the < 130 mmHg group (incidence, 0.18% person-year; 95% CI, 0.04–0.71) among 2627 patients with previous ischemic stroke taking either cilostazol or aspirin for a mean duration of 29 months. In sum, we suggest that stricter blood pressure control appears necessary for the prevention of hemorrhagic complications and that the systolic blood pressure criterion in the HAS-BLED scoring should ideally be changed from 160 mmHg to 130 mmHg.

The dose reduction criteria for NOACs (e.g., age, renal disorder, and body weight) were not met in any of our patients, indicating that the dose at initation of NOAC therapy was not excessive in any of our patients. It is possible that hemorrhage arises from pre-existing cerebral microbleeds (CMBs) [19]. However, magnetic resonance fast field echo imaging was not performed at the start of NOAC therapy and the presence of CMBs was therefore unclear in this study. Although concurrent antiplatelet and NOAC therapy is thought to induce hemorrhage, 2 of our patients who had received concurrent antiplatelet medication did not show hematoma expansion. None of the patients had hepatic and/or renal disorder or hemorrhage tendency and did not receive concurrent NSAIDs. Only 1 patient had a previous stroke.

Taken together, the risk for symptomatic ICH remained in patients on NOAC therapy, even when mean systolic blood pressure before onset was controlled to around 137 mmHg and the HAS-BLED score was ≤ 2. It is likely that stricter blood pressure control is necessary and prospective studies are necessary to investigate this issue further.

The limitations of this study include its retrospective and single-center design, short observation period, small number of cases, lack of comparison with warfarin-associated ICH cases, lack of more data in 24 h blood pressure before ICH onset, and absence of edoxaban cases (due to its later launch).

In conclusion, symptomatic ICH occurred early in NOAC therapy, and hematoma volume was small and remained so despite the lack of infusion of FFP, PCC, rFVIIa or hemodialysis. Our findings suggest that even stricter blood pressure lowering and control within the acceptable range may be advisable to prevent ICH during NOAC therapy. Further prospective studies are warranted.
